# Early glucose and sodium trajectories and short-term outcomes in extremely preterm infants

**DOI:** 10.3389/fped.2026.1878551

**Published:** 2026-08-05

**Authors:** Spyros Gialamas, Nima Naseh, Erik Groth, Karl Wilhelm Olsson, Johan Ågren, Richard Sindelar

**Affiliations:** 1 Department of Women’s and Children’s Health, Akademiska University Hospital, Uppsala University, Uppsala, Sweden; 2 Department of Surgical Sciences, Akademiska University Hospital, Uppsala University, Uppsala, Sweden

**Keywords:** bronchopulmonary dysplasia, extremely preterm infants, glucose, glycemic variability, neonatal outcomes, osmolarity, postnatal trajectories, sodium

## Abstract

**Introduction:**

Extremely preterm infants commonly develop marked early changes in plasma glucose and sodium concentrations, but their clinical relevance remains unclear. We therefore aimed to characterize plasma glucose, sodium, and calculated osmolarity, and examine their associations with short-term neonatal outcomes.

**Methods:**

We conducted a retrospective single-center cohort study of 42 extremely preterm infants (22_+0_ - 27_+6_ weeks’ gestation). Levels, variability, and temporal patterns in serial plasma glucose and sodium measurements, as well as calculated plasma osmolarity, were analyzed during the first postnatal week. Associations with neonatal outcomes were assessed in adjusted analyses.

**Results:**

The lowest glucose value and greater glucose variability during the first postnatal week were associated with duration of mechanical ventilation, bronchopulmonary dysplasia and the composite outcome of bronchopulmonary dysplasia or death. A larger decrease in glucose during the first 4 postnatal days was associated with necrotizing enterocolitis. Plasma sodium and calculated plasma osmolarity during the first postnatal week were associated with surgical ligation of patent ductus arteriosus but showed no consistent associations with the other outcomes studied.

**Discussion:**

These exploratory findings suggest that associations with subsequent neonatal morbidity were more consistently observed for glucose-derived measures than for plasma sodium or calculated plasma osmolarity. Whether these associations are causal or reflect underlying physiological vulnerability requires further investigation.

## 1 Introduction

Survival among extremely preterm infants has improved substantially over recent decades; however, major neonatal morbidities such as bronchopulmonary dysplasia (BPD), intraventricular hemorrhage (IVH), and retinopathy of prematurity (ROP) continue to affect a large proportion of survivors (1, 2). Infants born at the lowest gestational ages (GA) undergo profound physiological transitions immediately after birth, marked by physiological immaturity that predisposes them to early instability in glucose, fluid balance, and electrolytes (3). These disturbances are particularly prominent during the first week of life, due to insensible water loss, not fully established nutritional support, and limited capacity of immature regulatory mechanisms to adapt to ongoing clinical interventions (4).

Disturbances in glucose homeostasis are common in extremely preterm infants and include both hyperglycemia and hypoglycemia early after birth (5–7). Neonatal hyperglycemia has been associated with higher mortality and increased risks of IVH and ROP in extremely low birth weight cohorts, whereas associations with BPD or prolonged respiratory support appear less consistent (8–10). At the other end of the spectrum, severe or recurrent hypoglycemia has been linked to brain injury and neurodevelopmental impairment, underscoring the vulnerability of the immature brain to both low and high glucose exposures (6, 11, 12). Importantly, given the lack of consensus on harmful glucose thresholds across maturational stages in preterm infants, several studies suggest that trajectory-based measures of glycemic exposure may be more informative than isolated peak values in predicting adverse outcomes. This supports the need to characterize glycemic dysregulation across complementary dimensions such as level, dispersion, and early rates of change, rather than relying solely on threshold definitions (7, 12, 13).

Abnormalities in plasma sodium concentration are similarly prevalent in extremely preterm infants, with both hypo- and hypernatremia frequently observed during the early postnatal period (14). Early dysnatremia, particularly hypernatremia, has been associated with severe IVH (14–16), increased mortality (14), and adverse neurodevelopmental outcomes (17). Moreover, dysnatremia has been associated with a broader spectrum of prematurity-related morbidities, including ROP, patent ductus arteriosus (PDA), and BPD (14, 18). Serum sodium concentrations reflect dynamic interactions between fluid balance, renal maturation, and clinical management. Consequently, reliance on isolated values or binary cutoffs may overlook clinically relevant exposure patterns (18, 19). Consistent with this, Brandon et al. used repeated measures, including sodium variability and calculated osmolality, and found that higher sodium variability was associated with surgical necrotizing enterocolitis (NEC) and in-hospital mortality (19).

Calculated plasma osmotic concentration—often reported as osmolality but commonly derived from formulas that, more precisely, compute osmolarity—is determined primarily by sodium, glucose, and, in many formulas, urea. Calculated plasma osmolarity may be used as an index of osmotic status (20, 21). Despite its biological relevance, data on calculated plasma osmolality/osmolarity in neonates are limited, with most studies relying on single early measurements and focusing mainly on short-term outcomes such as in-hospital mortality (20). More broadly, the neonatal literature has tended to treat calculated osmolality/osmolarity as an absolute level rather than as time-varying exposure, and relatively few studies have evaluated trajectories or within-infant variability of calculated osmolality over time (19).

We therefore aimed to explore whether early changes in plasma glucose, sodium, and calculated plasma osmolarity were associated with short-term neonatal outcomes in extremely preterm infants, using multiple complementary exposure summaries reflecting level, variability, and temporal patterns, including both magnitude and rate of change.

## 2 Materials and methods

### 2.1 Study design and population

This retrospective cohort study included extremely preterm infants born at GA 22 + 0 to 27 + 6 weeks who were admitted to the neonatal intensive care unit (NICU) at Uppsala University Hospital within the first 12 h of life, between 1 June 2011 and 31 May 2012. Infants with chromosomal abnormalities or major congenital malformations were excluded. Because exposure measures were derived from serial measurements over the first 4–7 postnatal days, analyses were restricted to infants who survived the relevant exposure window. To minimize exposure contamination by major intercurrent events, infants who underwent abdominal surgery during the relevant exposure window were excluded from analyses using that window.

### 2.2 Data collection

Eligible infants were identified using institutional records and the Swedish Neonatal Quality Register (SNQ), with detailed clinical, laboratory, and outcome data extracted from medical records and institutional databases. Plasma sodium and glucose concentrations were obtained simultaneously as part of routine clinical care, using standardized laboratory panel analyses. Measurements were frequent during the first postnatal week, with higher sampling density in the earliest days of life, particularly among the most immature infants (Supplementary Figure S1).

### 2.3 Exposure definitions

Plasma osmolarity (mOsm/L) was calculated as 2 × [Na⁺] + [glucose] (mmol/L), omitting urea because it was not routinely available. Therefore, this index should be interpreted as a pragmatic plasma sodium- and glucose-based estimate of the dominant osmotic contributors rather than a direct substitute for measured plasma osmolality, which depends on the chosen formula and included solutes (21). Exposures were defined *a priori* to capture complementary aspects of early metabolic status for plasma sodium, glucose, and calculated plasma osmolarity, including absolute levels, variability, and temporal patterns. Exposure windows were defined based on outcome timing: Exposures for IVH and NEC were assessed during the first 4 postnatal days and for all other outcomes during the first 7 postnatal days. The 7-day window was chosen to capture the early postnatal sodium trajectory in extremely preterm infants, including the initial rise commonly observed during the first days after birth and the subsequent decline later in the first week (22).

### 2.4 Outcome definitions

The neonatal outcomes examined were IVH, PDA surgical ligation, NEC, ROP, BPD, death before 36 weeks' postmenstrual age (PMA), the composite outcome of BPD or death before 36 weeks' PMA, and duration of mechanical ventilation (MV). IVH was classified according to the Papile grading system, based on cranial ultrasound examinations independently reviewed by two pediatric radiologists, and analyzed as any IVH (grade ≥1) (23). PDA treatment was defined as surgical ligation of the ductus arteriosus during neonatal hospitalization. NEC was restricted to surgical cases (intraoperative findings consistent with the diagnosis, with or without histopathological confirmation) or postmortem pathological confirmation (24). ROP was assessed through serial screening examinations according to published screening recommendations and classified using international classification of retinopathy of prematurity (ICROP). ROP was analyzed as any ROP (stage ≥ 1) in either eye (25). BPD was defined as the requirement for supplemental oxygen at 36 weeks' PMA (26). Duration of MV was defined as the total number of days of invasive respiratory support until death or discharge.

### 2.5 Data analysis

Binary outcomes were analyzed using Firth penalized logistic regression to reduce small-sample bias and accommodate sparse outcome distributions; results are presented as odds ratios (ORs) with 95% confidence intervals (CIs). Because BPD status cannot be ascertained in infants who died before 36 weeks' PMA, BPD analyses were restricted to survivors to 36 weeks’ PMA, and we additionally analyzed the composite outcome of BPD or death before 36 weeks' PMA. Duration of MV was analyzed using negative binomial regression to account for overdispersion; results are presented as incidence rate ratios (IRRs) with 95% CIs. Because duration of MV is truncated by death, analyses including all infants were complemented by a sensitivity analysis restricted to infants surviving to discharge. Continuous exposures were scaled to predictor-appropriate, clinically meaningful increments. Maximum decrease measures were defined as the absolute magnitude of the largest decrease (and corresponding rate), such that higher values indicate larger decreases. For nadir measures (minimum values), associations were expressed per lower nadir by reporting reciprocal effect estimates (i.e., 1/OR or 1/IRR, equivalent to modeling the negative of the nadir). Primary analyses modeled continuous exposures linearly. Functional form was assessed by comparing linear models with natural cubic splines. For MV duration models, additional checks suggested some departures from strict log-linearity and influence sensitivity; therefore, IRRs were interpreted as average associations over the observed exposure range. Spline models used 3 df in adjusted Firth logistic models and 4 df in adjusted negative binomial MV duration models. Models were adjusted *a priori* for gestational age, birth weight z-score, and sex. Gestational age was entered as a standardized z-score, calculated using the mean and SD of gestational age in the full study cohort. For binary outcomes with fewer than 10 events, adjusted models were restricted to gestational age standardized z-score and birth weight z-score to reduce overfitting. Crude models and sensitivity analyses additionally accounting for maximum weight loss were also performed using window-matched maximum weight-loss variables; these results are reported in the Supplementary Material. Missing data were minimal; no imputation was performed, and models used complete-case data for variables included in each analysis. Hourly plasma sodium, glucose, and calculated plasma osmolarity values were linearly interpolated for descriptive visualization only and were not used in regression analyses. Given the number of exposure measures and outcomes examined, analyses were considered exploratory, with emphasis placed on coherent patterns across related measures rather than isolated *p*-values. Statistical analyses were developed and performed in collaboration with a statistician and critically discussed among the coauthors to ensure suitability and robustness in the context of the available sample size. Analyses were conducted in R (version 4.4.2; R Foundation for Statistical Computing, Vienna, Austria).

### 2.6 Ethics

The study was conducted in accordance with the World Medical Association Declaration of Helsinki, relevant Swedish legislation, and institutional guidelines. The study was approved by the Swedish Ethical Review Authority on 22 March 2017 (protocol number 2017/115, including amendment approvals 2017/115/1 and 2017/115/2). Owing to the retrospective design and the use of deidentified data, the Swedish Ethical Review Authority waived the requirement for informed parental consent. The privacy rights of the study participants were respected, and no personal or identifying information is included in the manuscript or Supplementary Material.

## 3 Results

A total of 57 infants with a GA of 22 + 0–27 + 6 weeks were admitted during the study period. Excluded infants were those admitted beyond 12 h (*n* = 6), discharged (*n* = 1), died (*n* = 6), or operated (*n* = 2) prior to day 4, leaving 42 infants for analysis. All included infants survived beyond postnatal day 7. Baseline characteristics and neonatal outcomes of the study cohort are presented in Table 1.

**Table 1:** Baseline characteristics and neonatal outcomes of the study cohort (*n* = 42).

Variable	Median (IQR) or *n* (%)
Maternal characteristics	
Age (years)	31.0 (28.0–36.0)
Parity	
1	19 (45.2)
2	13 (31.0)
3	4 (9.5)
4	5 (11.9)
5	1 (2.4)
Antenatal steroid administration	
<24 h before birth	7 (16.7)
>24 h before birth	35 (83.3)
Antenatal antibiotic administration^a^	39 (95.1)
Multiple pregnancy status	
Singleton	25 (59.6)
Twin	14 (33.3)
Triplet	3 (7.1)
Diabetes mellitus/gestational diabetes^b^	1 (2.7)
Preeclampsia	9 (21.4)
Chorioamnionitis	13 (31.0)
PPROM	10 (23.8)
Delivery method	
Vaginal birth	11 (26.2)
Cesarean section	31 (73.8)
Infant characteristics	
Gestational age (weeks)	25.6 (24.1–26.5)
Female sex	26 (61.9)
Birth weight (g)	759.0 (597.0–885.8)
Birth length (cm)	32.3 (30.0–34.0)
Birth head circumference (cm)	23.4 (22.0–24.5)
Small for gestational age	9 (21.4)
Apgar score (min)	
1 ^c^	5.0 (3.0–7.0)
5	7.0 (5.0–8.0)
10	8.0 (7.0–9.0)
Respiratory distress syndrome	41 (97.6)
Surfactant, number of doses	
0	1 (2.4)
1	29 (69.0)
2	11 (26.2)
3	1 (2.4)
Age at first surfactant dose (min)^d^	7.0 (4.0–8.0)
Antibiotic treatment during the first postnatal week	39 (92.9)
Neonatal outcomes	
IVH, any grade	11 (26.2)
PDA surgical ligation	5 (11.9)
NEC^e^	6 (14.3)
ROP, any stage	18 (42.9)
BPD^f^	18 (52.9)
BPD or death before 36w	26 (61.9)
Death before 36w	8 (19.0)
Duration of MV (days)^g^	17.0 (4.0–49.8)

a Missing for one infant.

b Missing for five infants.

c Missing for one infant.

d Missing for three infants.

e Defined as surgery-requiring NEC with intraoperative findings consistent with the disease (± histopathological confirmation) or NEC confirmed at postmortem pathological examination.

f Among 34 infants surviving to 36w.

g Until death or discharge.

36w, 36 weeks’ postmenstrual age; BPD, bronchopulmonary dysplasia; IQR, interquartile range; IVH, intraventricular hemorrhage; MV, mechanical ventilation; NEC, necrotizing enterocolitis; PDA, patent ductus arteriosus; PPROM, preterm prelabor rupture of membranes; ROP, retinopathy of prematurity.

**Figure 1:**
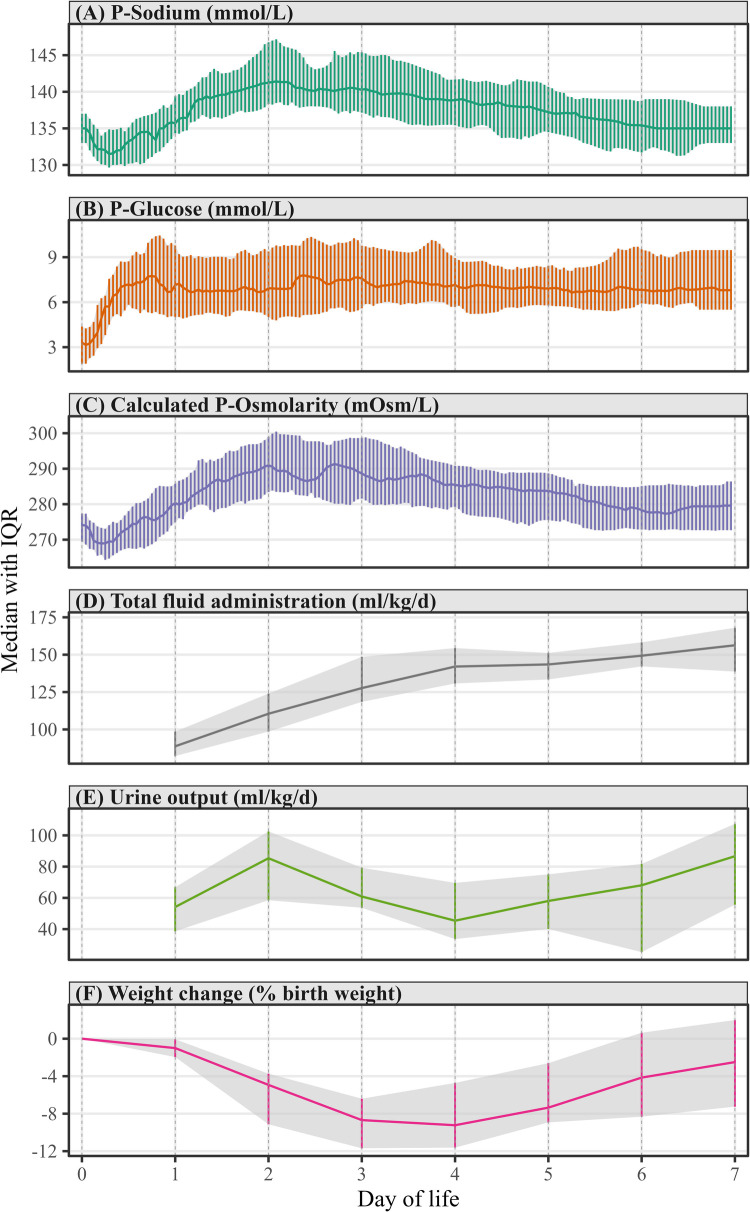
Postnatal trajectories during the first postnatal week in the full cohort (n=42). Solid lines show medians and shaded areas show IQRs for hourly plasma sodium **(A)**, plasma glucose **(B)**, and calculated plasma osmolarity **(C)**, and for daily total fluid administration **(D)**, urine output **(E)**, and weight change **(F)**. Hourly plasma sodium and glucose values were linearly interpolated between measurements; plasma osmolarity was calculated using interpolated plasma sodium and glucose. Daily variables are shown from day 1 onward. For total fluid administration and urine output, the day 1 values summarize the first 24 postnatal hours; weight change is shown at daily time points.

Figure 1 shows hourly interpolated median trajectories with interquartile ranges (IQRs) for plasma sodium, glucose, and calculated plasma osmolarity during the first postnatal week. Plasma sodium and calculated plasma osmolarity displayed a brief early fall, followed by a rise to a peak around days 2–3, and then a gradual decline. In contrast, glucose showed a brief early fall, followed by a rapid increase, and then remained relatively stable. Trajectories of ,uid administration, urine output, and weight change over the same period are also shown. Patterns were broadly similar across GA groups, although lower-GA infants tended to have higher median values; these comparisons were descriptive and not formally tested (Supplementary Figure S2). These patterns were consistent with those observed in the raw, non-interpolated measurements (data not shown).

Adjusted associations between early plasma sodium, glucose, and calculated plasma osmolarity exposure variables and neonatal outcomes are summarized in Table 2, with exact *p*-values provided in Supplementary Table S1. A lower glucose nadir during the first postnatal week was associated with longer duration of MV, BPD, and the composite outcome of BPD or death before 36 weeks' PMA. Several additional measures reflecting glucose instability, including larger glucose decreases and greater glucose variability, were also associated with respiratory outcomes. A larger maximum glucose decrease during the first 4 postnatal days was additionally associated with NEC. In contrast, plasma sodium- and calculated plasma osmolarity-derived measures showed inverse associations with PDA surgical ligation but no consistent associations with IVH, NEC, ROP, respiratory outcomes, or death.

**Table 2:** Adjusted associations of early plasma sodium, glucose, and calculated osmolarity measures with neonatal outcomes.

Outcome (events/*N*)	IVH (11/42)	PDA surgical ligation (5/41)	NEC (6/42)	ROP (17/41)	BPD (17/33)	BPD/death before 36w (25/41)	Death before 36w (8/41)	Duration of MV (*N* = 41)
Exposure measure	OR (95% CIs)	OR (95% CIs)	OR (95% CIs)	OR (95% CIs)	OR (95% CIs)	OR (95% CIs)	OR (95% CIs)	IRR (95% CIs)
*P*-sodium								
Median	0.47 (0.17–1.13)	1.19 (0.28–6.36)	0.56 (0.17–1.80)	1.87 (0.70–5.89)	2.21 (0.56–11.82)	0.90 (0.31–2.61)	0.35 (0.09–1.10)	0.88 (0.57–1.38)
Peak	0.98 (0.50–1.86)	0.39 (0.08–1.06)	0.93 (0.41–1.90)	1.48 (0.81–2.94)	1.55 (0.66–4.25)	1.51 (0.70–3.55)	1.40 (0.71–2.87)	0.91 (0.69–1.21)
Nadir	0.81 (0.32–1.85)	0.49 (0.10–1.61)	1.14 (0.38–3.05)	0.65 (0.27–1.44)	0.78 (0.25–2.20)	0.98 (0.38–2.60)	1.21 (0.46–3.12)	0.99 (0.68–1.44)
Range peak–nadir	0.87 (0.45–1.59)	**0.16** **(0.02**–**0.58)**	0.98 (0.45–1.97)	1.14 (0.64–2.08)	1.22 (0.59–2.68)	1.39 (0.70–3.05)	1.50 (0.77–3.09)	0.90 (0.68–1.19)
Max *Δ*48 h	0.91 (0.40–1.87)	**0.17** (**0.02**–**0.82)**	1.11 (0.44–2.56)	1.21 (0.61–2.48)	1.05 (0.44–2.66)	1.13 (0.49–2.74)	1.16 (0.50–2.54)	0.83 (0.60–1.15)
Max increase	0.79 (0.41–1.42)	**0.26** (**0.04**–**0.81)**	0.82 (0.36–1.64)	1.22 (0.71–2.14)	1.34 (0.69–2.76)	1.43 (0.78–2.82)	1.40 (0.74–2.74)	0.91 (0.71–1.18)
Rate max increase	1.02 (0.60–1.40)	1.02 (0.49–1.28)	1.30 (0.94–2.30)	0.92 (0.44–1.16)	0.61 (0.24–1.34)	1.00 (0.79–1.33)	1.14 (0.91–1.51)	1.01 (0.89–1.13)
Max decrease	1.50 (0.71–3.24)	0.43 (0.11–1.13)	1.35 (0.54–3.23)	0.96 (0.51–1.83)	0.76 (0.30–1.82)	0.98 (0.45–2.16)	1.60 (0.78–3.57)	1.06 (0.78–1.42)
Rate max decrease	0.90 (0.61–1.03)	0.73 (0.07–1.16)	0.96 (0.64–1.07)	1.00 (0.82–1.18)	1.08 (0.88–1.30)	1.07 (0.87–1.30)	1.02 (0.79–1.22)	0.94 (0.86–1.03)
*P*-glucose								
Median	1.13 (0.87–1.51)	0.92 (0.58–1.33)	1.06 (0.77–1.45)	1.26 (0.93–1.80)	1.13 (0.77–1.71)	1.07 (0.70–1.63)	0.96 (0.67–1.34)	1.06 (0.92–1.22)
Peak	1.06 (0.92–1.27)	0.96 (0.77–1.10)	1.14 (0.97–1.41)	1.01 (0.92–1.11)	1.07 (0.94–1.30)	1.09 (0.96–1.32)	1.08 (0.97–1.21)	1.03 (0.99–1.08)
Nadir	1.18 (0.67–2.22)	1.44 (0.61–4.57)	1.24 (0.60–2.95)	0.94 (0.53–1.68)	**3.24** (**1.32**–**15.11)**	**4.41** (**1.69**–**23.22)**	2.14 (0.94–6.91)	**1.64** (**1.28**–**2.09)**
Range peak–nadir	1.07 (0.93–1.26)	0.97 (0.80–1.10)	1.14 (0.97–1.39)	1.01 (0.92–1.11)	1.13 (0.97–1.45)	1.18 (1.00–1.50)	1.09 (0.99–1.22)	1.04 (1.00–1.09)
Max *Δ*48 h	1.06 (0.92–1.25)	0.96 (0.78–1.12)	1.14 (0.97–1.38)	1.02 (0.91–1.14)	1.22 (1.00–1.60)	**1.26** (**1.04**–**1.68)**	1.10 (0.97–1.26)	**1.06** (**1.01**–**1.11)**
Max increase	1.06 (0.92–1.24)	0.98 (0.81–1.11)	1.13 (0.97–1.37)	1.01 (0.92–1.11)	1.14 (0.97–1.46)	1.18 (1.00–1.51)	1.09 (0.98–1.22)	1.04 (1.00–1.09)
Rate max increase	1.01 (0.94–1.08)	1.05 (0.95–1.15)	1.03 (0.93–1.12)	1.03 (0.96–1.11)	1.06 (0.97–1.18)	1.04 (0.96–1.15)	1.00 (0.91–1.08)	1.02 (0.99–1.05)
Max decrease	1.15 (0.96–1.44)	1.03 (0.82–1.29)	**1.34** (**1.07**–**1.86)**	1.05 (0.89–1.24)	**1.33** (**1.02**–**1.92)**	**1.36** (**1.06**–**1.94)**	1.17 (0.96–1.52)	**1.08** (**1.00**–**1.17)**
Rate max decrease	1.00 (0.92–1.08)	0.92 (0.64–1.07)	1.07 (0.99–1.16)	0.92 (0.77–1.03)	1.19 (0.96–1.96)	1.28 (0.99–1.95)	1.09 (0.99–1.23)	1.01 (0.97–1.06)
Calculated P-osmolarity								
Median	0.60 (0.26–1.27)	0.70 (0.19–2.92)	0.62 (0.22–1.65)	2.37 (0.89–7.78)	2.87 (0.73–15.91)	0.99 (0.37–2.76)	0.38 (0.11–1.09)	0.94 (0.62–1.43)
Peak	0.96 (0.54–1.69)	0.50 (0.17–1.12)	1.07 (0.55–2.04)	1.37 (0.78–2.56)	1.65 (0.74–4.23)	1.67 (0.81–3.78)	1.61 (0.84–3.46)	0.99 (0.76–1.28)
Nadir	0.70 (0.26–1.65)	0.79 (0.22–2.34)	0.96 (0.29–2.68)	0.74 (0.32–1.62)	0.89 (0.29–2.57)	1.02 (0.39–2.72)	0.96 (0.36–2.40)	1.09 (0.75–1.57)
Range peak–nadir	0.83 (0.46–1.43)	**0.21** (**0.04**–**0.72)**	1.05 (0.53–1.98)	1.19 (0.66–2.26)	1.51 (0.70–3.58)	1.58 (0.81–3.45)	1.68 (0.84–3.76)	1.02 (0.77–1.36)
Max *Δ*48 h	0.82 (0.39–1.53)	0.31 (0.04–1.22)	0.96 (0.41–1.97)	1.24 (0.67–2.47)	1.38 (0.59–3.73)	1.54 (0.73–4.16)	1.57 (0.77–3.30)	1.04 (0.77–1.40)
Max increase	0.80 (0.44–1.35)	**0.34** (**0.08**–**0.95)**	0.94 (0.46–1.75)	1.18 (0.69–2.11)	1.66 (0.83–3.70)	1.82 (0.97–3.90)	1.85 (0.94–4.08)	1.03 (0.80–1.34)
Rate max increase	1.18 (0.54–2.53)	1.06 (0.34–2.56)	1.17 (0.44–2.82)	0.89 (0.41–1.80)	0.50 (0.15–1.24)	0.43 (0.12–1.09)	0.74 (0.23–1.71)	0.98 (0.70–1.37)
Max decrease	1.23 (0.57–2.61)	0.59 (0.18–1.51)	1.69 (0.68–4.35)	1.09 (0.58–2.07)	0.75 (0.31–1.65)	0.85 (0.40–1.75)	1.43 (0.69–3.06)	1.01 (0.75–1.36)
Rate max decrease	0.92 (0.66–1.04)	0.87 (0.37–1.27)	0.72 (0.33–1.03)	0.94 (0.66–1.19)	0.98 (0.62–1.33)	0.94 (0.55–1.33)	0.89 (0.39–1.21)	0.92 (0.81–1.05)

Bold indicates 95% CI excluding 1.0. Exact *p*-values for crude and adjusted models are provided in Supplementary Tables S1, S2.

Exposure windows were defined as the first 4 postnatal days for IVH and NEC, and the first 7 postnatal days for all other outcomes. One infant was excluded from 7-day exposure analyses due to abdominal surgery on postnatal day 5.

Within each window, measures were defined as median, peak, nadir, range peak–nadir (absolute difference between highest and lowest values), max *Δ*48 h (largest absolute difference between any two values ≤48 h apart), max increase (largest increase to a later peak) and corresponding rate per day, and max decrease (largest decrease to a later nadir) and corresponding rate per day.

ORs are estimated using Firth penalized logistic regression and IRRs using negative binomial regression. Estimates are scaled per 5 mmol/L for plasma sodium, 1 mmol/L for plasma glucose, and 10 mOsm/L for calculated osmolarity and per corresponding units/day for rate measures; nadir effects are expressed per lower nadir (reciprocal of the modeled per-higher-nadir effect), and max decrease variables are magnitudes, so higher values indicate larger decreases.

For MV duration, IRRs assume linearity on the log scale; where diagnostics suggest non-linearity and/or influence sensitivity, IRRs reflect average associations over the observed range.

Plasma osmolarity was calculated as 2 × [Na⁺] + glucose (mOsm/L), using analyte concentrations in mmol/L. Models were adjusted for gestational age entered as a standardized z-score (calculated using the mean and SD of gestational age in the full study cohort), birth weight z-score, and sex; for outcomes with <10 events (PDA surgical ligation, NEC and death before 36w), adjusted models were restricted to gestational age z-score and birth weight z-score to reduce overfitting.

IVH is reported as any grade; ROP as any stage; NEC as surgically or postmortem confirmed; BPD is defined at 36w among survivors. Duration of MV is measured in days and calculated from birth until discharge or death, whichever occurred first.

36w, 36 weeks’ postmenstrual age; BPD, bronchopulmonary dysplasia; CI, confidence interval; IRR, incidence rate ratio; IVH, intraventricular hemorrhage; MV, mechanical ventilation; NEC, necrotizing enterocolitis; OR, odds ratio; PDA, patent ductus arteriosus; ROP, retinopathy of prematurity.

Compared with crude models (Supplementary Table S2), effect estimates were generally attenuated after adjustment for glucose-level measures and several calculated plasma osmolarity measures, and some associations no longer met conventional levels of statistical significance. Nevertheless, the associations between lower glucose nadir and respiratory outcomes remained robust. Sensitivity analyses demonstrated a similar overall pattern of findings. Restriction to survivors to discharge attenuated most associations, although a lower glucose nadir remained associated with longer MV (Supplementary Table S3). After additional adjustment for maximum percentage weight loss, the main glucose-related associations were preserved, and additional associations with death before 36 weeks' PMA emerged (Supplementary Table S4).

## 4 Discussion

In this cohort of extremely preterm infants, we characterized early postnatal plasma sodium, glucose, and calculated plasma osmolarity across complementary dimensions of level, variability, and temporal patterns. Descriptive trajectories showed that plasma sodium and calculated plasma osmolarity decreased during the first postnatal day, then rose to a peak around days 2–3, and then gradually declined. In contrast, glucose exhibited a brief early fall followed by a rapid increase and subsequent relative stabilization. In adjusted analyses, lower glucose nadir and several measures of early glucose instability were associated primarily with respiratory outcomes and NEC. In contrast, sodium- and calculated plasma osmolarity-derived measures were associated only with PDA surgical ligation and showed no consistent associations with the remaining neonatal outcomes.

To our knowledge, prior studies have mainly presented longitudinal fluid balance and sodium trajectories (22, 27) or have focused on single analytes rather than integrated multianalyte profiles (17, 19, 20). Our data complement these studies by providing higher–temporal–resolution visualizations of early plasma sodium, glucose, and calculated osmolarity alongside concurrent changes in fluid balance, displayed on an hourly grid using linear interpolation between observed measurements. The observed covariation of early weight loss and rising urine output with evolving sodium and osmolarity trajectories is compatible with the early postnatal transition in fluid balance, including contraction of extracellular water (22, 27).

Prior studies in extremely and very preterm infants have largely framed neonatal dysglycemia in terms of hyperglycemia, which is common and has repeatedly been associated with mortality and short-term morbidity in observational studies (5, 7–10, 28). However, heterogeneous exposure definitions, variation in treatment practices, and confounding by immaturity, illness severity, and nutrition complicate interpretation (5, 7, 10, 28). Direct evidence regarding low glucose nadir and early glycemic instability is more limited (29). In our cohort, these measures appeared more informative than isolated hyperglycemic peaks, particularly for respiratory morbidity. Because BPD is assessed only among infants surviving to 36 weeks' PMA, the composite outcome of BPD/death before 36 weeks' PMA may provide a more robust respiratory endpoint in this setting. Earlier reports have linked higher glucose levels to ROP, but this association was not maintained in our adjusted analyses (13).

Conceptually, our findings support a view of neonatal dysglycemia that extends beyond isolated hyperglycemic peaks and may be better captured by a low glucose nadir and early glucose instability (6, 11, 29). This does not establish causality; rather, these measures may reflect immaturity, limited metabolic reserves, illness severity, and immunological vulnerability in extremely preterm infants (6, 11, 29). Experimental and translational work nevertheless provides biological plausibility for links between dysglycemia, inflammation, and organ injury, particularly in the immature lung and possibly the gut (30–35). Nonetheless, given the observational design of our study, dysglycemia should be interpreted primarily as a marker of early systemic stress and physiological instability rather than a proven causal driver.

In previous studies, excess sodium exposure, dysnatremia, and early sodium variability have been linked to adverse outcomes in extremely preterm infants, including severe IVH, mortality, and adverse neurodevelopment (4, 14–19, 36). Several reports have also suggested that sodium load or intake may be more informative than plasma sodium concentrations alone (15, 18, 36). By contrast, evidence relating measured plasma osmolality or calculated osmotic indices to neonatal outcomes is limited and largely derives from heterogeneous NICU cohorts assessed at admission, reducing comparability with first-week trajectories of calculated plasma osmolarity in extremely preterm infants (20). Liu et al. reported a U-shaped association between admission plasma osmolality and in-hospital mortality (20), and Brandon et al. found that increasing sodium variability during the first 96 h was associated with surgical NEC and in-hospital mortality, while also noting that serum sodium and calculated osmolality were poor proxies for percent weight change (19). In our cohort, plasma sodium- and calculated osmolarity-based measures showed no consistent adjusted associations with major neonatal outcomes, apart from inverse associations of greater variability or larger increase with PDA surgical ligation.

Taken together, prior studies and our findings provide little support for osmolarity being a direct mediator of short-term morbidity or mortality. The lack of consistent associations for plasma sodium and calculated plasma osmolarity may reflect the fact that plasma sodium concentration does not fully capture sodium load or net fluid balance, and that calculated plasma osmolarity may introduce additional measurement errors rather than encompassing clinically relevant effects. The lack of association between sodium- or osmolarity-based changes and IVH in our cohort may partly reflect lower underlying IVH risk in a cohort with high antenatal corticosteroid exposure (37). As the need for subsequent PDA surgical ligation is influenced by many factors, including ductal pathophysiology, clinical course, local treatment strategies, and survival to later intervention, the inverse association with early changes in plasma sodium should be interpreted cautiously. Moreover, no clear physiological mechanism readily explains why greater sodium variability would reduce the need for surgical ligation. The corresponding calculated plasma osmolarity findings largely paralleled rather than strengthened the sodium findings, suggesting that the glucose component did not markedly potentiate the association between sodium and PDA surgery. Therefore, these isolated findings may reflect chance or residual confounding rather than causal relationships.

The differing association patterns observed for plasma glucose, plasma sodium, and calculated plasma osmolarity should not be interpreted as evidence that one metabolic domain is inherently more important than another. Rather, they may reflect distinct underlying physiological processes and different relationships with specific neonatal outcomes. Given the exploratory design, modest sample size, and multiple comparisons, these observations should be regarded as hypothesis-generating and require confirmation in larger prospective studies.

A key strength of this study is the detailed characterization of early plasma sodium, glucose, and calculated plasma osmolarity across complementary dimensions, rather than relying on single-threshold definitions alone. The trajectory visualizations provided higher temporal granularity than prior reports and highlighted a brief initial fall in plasma sodium before the subsequent rise, a pattern that has been less apparent in the literature (19, 22, 27). In addition, we used prespecified covariate adjustment and modeling approaches appropriate for a small cohort with sparse events, presenting adjusted estimates alongside crude estimates and sensitivity analyses.

Several limitations should be considered. The cohort was modest in size and from a single center, which may reduce statistical power and limit generalizability. Furthermore, the cohort reflects clinical practice from 2011 to 2012. Since then, several aspects of neonatal care—including respiratory support, nutritional management, and PDA treatment strategies—have evolved. These changes may influence both metabolic trajectories and outcome risks, thereby limiting the generalizability of our findings to current practice. At the same time, blood sampling practices have become more restrictive to minimize iatrogenic blood loss and painful procedures. The relatively high sampling density in this cohort, particularly during the first postnatal days (Supplementary Figure S1), enabled a more detailed characterization of early glucose, sodium, and calculated plasma osmolarity trajectories than would likely be feasible under current routines. The attenuation of several crude associations after adjustment suggests confounding by infant maturity and size, and residual confounding by correlated, time-varying illness severity, including infection, inflammation, and therapeutic factors. Because of sparse events, some adjusted models were necessarily parsimonious, which may have left residual confounding. Individual-level data regarding insulin administration were unavailable. However, insulin therapy was not part of routine practice at our unit during the study period, and therefore confounding by glucose-lowering treatment was likely limited. Detailed individual-level data on sodium and glucose intake were unavailable, although standardized NICU protocols limited practice variation, and total fluid administration is shown descriptively in Figure 1. The trajectory plots are descriptive and rely on linear interpolation between observed measurements, which may smooth short-lived extremes. Furthermore, calculated osmolarity was not directly measured and omitted urea and other minor osmoles; thus, it represents an approximation of effective osmotic concentration (tonicity) rather than total measured plasma osmolality. Formula-related error and collinearity with its component variables may also have attenuated associations. In addition, analyses of MV duration restricted to survivors should be interpreted as conditional on survival. Because infants who died early could not contribute complete early trajectories, survival-related selection may also have influenced exposure–outcome associations. Finally, given the number of exposure measures and outcomes, some observed associations may reflect chance, and emphasis should therefore be placed on coherent patterns across related measures rather than isolated findings.

In conclusion, in this cohort of extremely preterm infants, early plasma glucose measures, particularly a lower glucose nadir and greater glycemic instability, were associated with subsequent respiratory morbidity and NEC. These exploratory findings suggest that early dysglycemia may serve as a marker of systemic stress and physiologic vulnerability, supporting a move beyond single-threshold definitions toward a more multidimensional characterization of early metabolic trajectories. Plasma sodium and calculated plasma osmolarity showed no consistent pattern of associations after adjustment, apart from an inverse association with PDA surgical ligation. Larger prospective studies are needed to clarify causality and clinical relevance.

## Data Availability

The data are available from the corresponding author upon reasonable request and subject to appropriate ethical, legal, and institutional approval.
