# Supplementary material for: Early glucose and sodium trajectories and short-term outcomes in extremely preterm infants
Source: Front Pediatr. 2026 Aug 5;14:1878551. doi: 10.3389/fped.2026.1878551 (PMC13485914; doi:10.3389/fped.2026.1878551)
Supplement: Supplementary file 1 [file Supplementaryfile1.pdf]

Supplementary Material

1 Supplementary Figures and Tables

1.1 Supplementary Figures

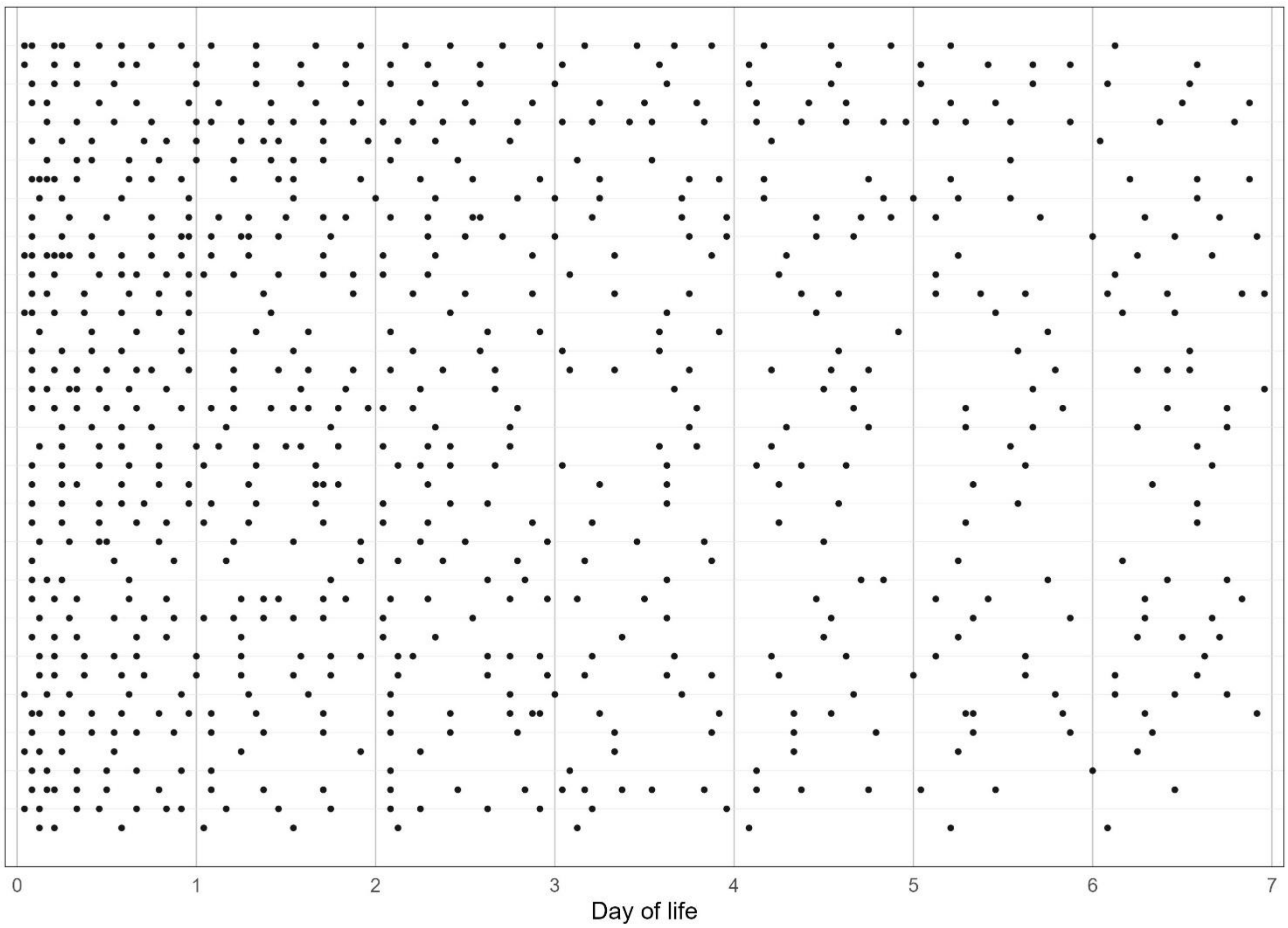

Supplementary Figure S1. Raster plot of plasma sodium and glucose sampling during the first postnatal week in the full cohort (n=42). Each row represents one infant, and each dot represents a blood sample. Infants are ordered by gestational age, with the most preterm infants shown at the top.

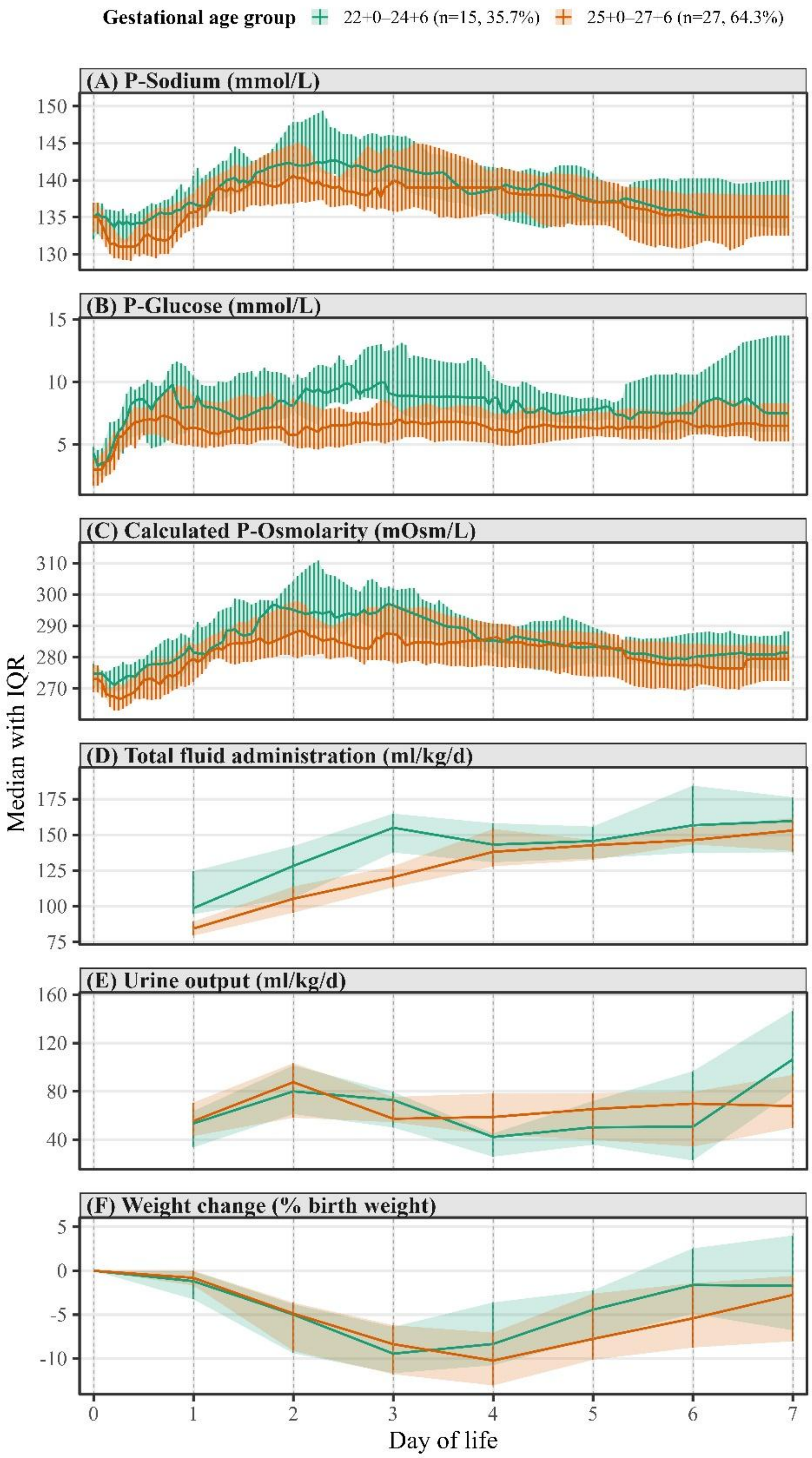

Supplementary Figure S2. Postnatal trajectories during the first postnatal week stratified by gestational age group: 22+0–24+6 weeks and 25+0–27+6 weeks. Solid lines show medians and shaded areas show IQRs for hourly plasma sodium (A), plasma glucose (B), and calculated plasma osmolarity (C), and for daily total fluid administration (D), urine output (E), and weight change (F). Hourly plasma sodium and glucose values were linearly interpolated between measurements; plasma osmolarity was calculated using interpolated plasma sodium and glucose.

1.2 Supplementary Tables

Supplementary Table S1. Adjusted associations of early plasma sodium, glucose, and calculated osmolarity measures with neonatal outcomes, including exact p-values.

Outcome (events/N)	IVH (11/42)		PDA surgical ligation (5/41)		NEC (6/42)		ROP (17/41)		BPD (17/33)		BPD/death before 36w (25/41)		Death before 36w (8/41)		Duration of MV (N=41)	
Exposure measure	OR (95% CIs)	p	OR (95% CIs)	p	OR (95% CIs)	p	OR (95% CIs)	p	OR (95% CIs)	p	OR (95% CIs)	p	OR (95% CIs)	p	IRR (95% CIs)	p
P-Sodium																
Median	0.47 (0.17 - 1.13)	0.092	1.19 (0.28 - 6.36)	0.827	0.56 (0.17 - 1.80)	0.328	1.87 (0.70 - 5.89)	0.217	2.21 (0.56 - 11.82)	0.261	0.90 (0.31 - 2.61)	0.842	0.35 (0.09 - 1.10)	0.071	0.88 (0.57 - 1.38)	0.585
Peak	0.98 (0.50 - 1.86)	0.945	0.39 (0.08 - 1.06)	0.068	0.93 (0.41 - 1.90)	0.836	1.48 (0.81 - 2.94)	0.209	1.55 (0.66 - 4.25)	0.333	1.51 (0.70 - 3.55)	0.298	1.40 (0.71 - 2.87)	0.330	0.91 (0.69 - 1.21)	0.515
Nadir	0.81 (0.32 - 1.85)	0.632	0.49 (0.10 - 1.61)	0.254	1.14 (0.38 - 3.05)	0.804	0.65 (0.27 - 1.44)	0.288	0.78 (0.25 - 2.20)	0.637	0.98 (0.38 - 2.60)	0.964	1.21 (0.46 - 3.12)	0.693	0.99 (0.68 - 1.44)	0.961
Range peak-nadir	0.87 (0.45 - 1.59)	0.660	0.16 (0.02 - 0.58)	**0.003**	0.98 (0.45 - 1.97)	0.945	1.14 (0.64 - 2.08)	0.660	1.22 (0.59 - 2.68)	0.591	1.39 (0.70 - 3.05)	0.348	1.50 (0.77 - 3.09)	0.227	0.90 (0.68 - 1.19)	0.469
Max Δ48 h	0.91 (0.40 - 1.87)	0.808	0.17 (0.02 - 0.82)	**0.023**	1.11 (0.44 - 2.56)	0.803	1.21 (0.61 - 2.48)	0.588	1.05 (0.44 - 2.66)	0.904	1.13 (0.49 - 2.74)	0.775	1.16 (0.50 - 2.54)	0.705	0.83 (0.60 - 1.15)	0.268
Max increase	0.79 (0.41 - 1.42)	0.445	0.26 (0.04 - 0.81)	**0.016**	0.82 (0.36 - 1.64)	0.584	1.22 (0.71 - 2.14)	0.476	1.34 (0.69 - 2.76)	0.388	1.43 (0.78 - 2.82)	0.253	1.40 (0.74 - 2.74)	0.301	0.91 (0.71 - 1.18)	0.479
Rate max increase	1.02 (0.60 - 1.40)	0.909	1.02 (0.49 - 1.28)	0.859	1.30 (0.94 - 2.30)	0.110	0.92 (0.44 - 1.16)	0.530	0.61 (0.24 - 1.34)	0.216	1.00 (0.79 - 1.33)	0.970	1.14 (0.91 - 1.51)	0.221	1.01 (0.89 - 1.13)	0.909
Max decrease	1.50 (0.71 - 3.24)	0.275	0.43 (0.11 - 1.13)	0.090	1.35 (0.54 - 3.23)	0.504	0.96 (0.51 - 1.83)	0.909	0.76 (0.30 - 1.82)	0.537	0.98 (0.45 - 2.16)	0.968	1.60 (0.78 - 3.57)	0.201	1.06 (0.78 - 1.42)	0.722
Rate max decrease	0.90 (0.61 - 1.03)	0.152	0.73 (0.07 - 1.16)	0.323	0.96 (0.64 - 1.07)	0.505	1.00 (0.82 - 1.18)	0.980	1.08 (0.88 - 1.30)	0.442	1.07 (0.87 - 1.30)	0.495	1.02 (0.79 - 1.22)	0.814	0.94 (0.86 - 1.03)	0.171
P-Glucose																
Median	1.13 (0.87 - 1.51)	0.369	0.92 (0.58 - 1.33)	0.654	1.06 (0.77 - 1.45)	0.695	1.26 (0.93 - 1.80)	0.136	1.13 (0.77 - 1.71)	0.516	1.07 (0.70 - 1.63)	0.738	0.96 (0.67 - 1.34)	0.830	1.06 (0.92 - 1.22)	0.433
Peak	1.06 (0.92 - 1.27)	0.402	0.96 (0.77 - 1.10)	0.572	1.14 (0.97 - 1.41)	0.124	1.01 (0.92 - 1.11)	0.789	1.07 (0.94 - 1.30)	0.340	1.09 (0.96 - 1.32)	0.205	1.08 (0.97 - 1.21)	0.138	1.03 (0.99 - 1.08)	0.157
Nadir	1.18 (0.67 - 2.22)	0.579	1.44 (0.61 - 4.57)	0.436	1.24 (0.60 - 2.95)	0.579	0.94 (0.53 - 1.68)	0.842	3.24 (1.32 - 15.11)	**0.006**	4.41 (1.69 - 23.22)	**0.001**	2.14 (0.94 - 6.91)	0.073	1.64 (1.28 - 2.09)	**<0.001**
Range peak-nadir	1.07 (0.93 - 1.26)	0.343	0.97 (0.80 - 1.10)	0.696	1.14 (0.97 - 1.39)	0.108	1.01 (0.92 - 1.11)	0.820	1.13 (0.97 - 1.45)	0.135	1.18 (1.00 - 1.50)	0.051	1.09 (0.99 - 1.22)	0.089	1.04 (1.00 - 1.09)	0.056
Max Δ48 h	1.06 (0.92 - 1.25)	0.398	0.96 (0.78 - 1.12)	0.665	1.14 (0.97 - 1.38)	0.124	1.02 (0.91 - 1.14)	0.718	1.22 (1.00 - 1.60)	0.050	1.26 (1.04 - 1.68)	**0.017**	1.10 (0.97 - 1.26)	0.127	1.06 (1.01 - 1.11)	**0.022**
Max increase	1.06 (0.92 - 1.24)	0.421	0.98 (0.81 - 1.11)	0.730	1.13 (0.97 - 1.37)	0.125	1.01 (0.92 - 1.11)	0.851	1.14 (0.97 - 1.46)	0.122	1.18 (1.00 - 1.51)	0.050	1.09 (0.98 - 1.22)	0.099	1.04 (1.00 - 1.09)	0.055
Rate max increase	1.01 (0.94 - 1.08)	0.785	1.05 (0.95 - 1.15)	0.330	1.03 (0.93 - 1.12)	0.542	1.03 (0.96 - 1.11)	0.363	1.06 (0.97 - 1.18)	0.229	1.04 (0.96 - 1.15)	0.327	1.00 (0.91 - 1.08)	0.971	1.02 (0.99 - 1.05)	0.234
Max decrease	1.15 (0.96 - 1.44)	0.138	1.03 (0.82 - 1.29)	0.789	1.34 (1.07 - 1.86)	**0.009**	1.05 (0.89 - 1.24)	0.555	1.33 (1.02 - 1.92)	**0.036**	1.36 (1.06 - 1.94)	**0.014**	1.17 (0.96 - 1.52)	0.117	1.08 (1.00 - 1.17)	**0.040**
Rate max decrease	1.00 (0.92 - 1.08)	0.962	0.92 (0.64 - 1.07)	0.385	1.07 (0.99 - 1.16)	0.091	0.92 (0.77 - 1.03)	0.172	1.19 (0.96 - 1.96)	0.169	1.28 (0.99 - 1.95)	0.077	1.09 (0.99 - 1.23)	0.077	1.01 (0.97 - 1.06)	0.652
Calculated P-Osmolarity																
Median	0.60 (0.26 - 1.27)	0.182	0.70 (0.19 - 2.92)	0.603	0.62 (0.22 - 1.65)	0.341	2.37 (0.89 - 7.78)	0.089	2.87 (0.73 - 15.91)	0.135	0.99 (0.37 - 2.76)	0.986	0.38 (0.11 - 1.09)	0.072	0.94 (0.62 - 1.43)	0.775
Peak	0.96 (0.54 - 1.69)	0.881	0.50 (0.17 - 1.12)	0.097	1.07 (0.55 - 2.04)	0.835	1.37 (0.78 - 2.56)	0.271	1.65 (0.74 - 4.23)	0.230	1.67 (0.81 - 3.78)	0.168	1.61 (0.84 - 3.46)	0.157	0.99 (0.76 - 1.28)	0.912
Nadir	0.70 (0.26 - 1.65)	0.424	0.79 (0.22 - 2.34)	0.678	0.96 (0.29 - 2.68)	0.946	0.74 (0.32 - 1.62)	0.451	0.89 (0.29 - 2.57)	0.827	1.02 (0.39 - 2.72)	0.970	0.96 (0.36 - 2.40)	0.934	1.09 (0.75 - 1.57)	0.654
Range peak-nadir	0.83 (0.46 - 1.43)	0.513	0.21 (0.04 - 0.72)	**0.010**	1.05 (0.53 - 1.98)	0.873	1.19 (0.66 - 2.26)	0.557	1.51 (0.70 - 3.58)	0.299	1.58 (0.81 - 3.45)	0.186	1.68 (0.84 - 3.76)	0.148	1.02 (0.77 - 1.36)	0.870
Max Δ48 h	0.82 (0.39 - 1.53)	0.538	0.31 (0.04 - 1.22)	0.113	0.96 (0.41 - 1.97)	0.917	1.24 (0.67 - 2.47)	0.490	1.38 (0.59 - 3.73)	0.468	1.54 (0.73 - 4.16)	0.269	1.57 (0.77 - 3.30)	0.211	1.04 (0.77 - 1.40)	0.814
Max increase	0.80 (0.44 - 1.35)	0.405	0.34 (0.08 - 0.95)	**0.039**	0.94 (0.46 - 1.75)	0.855	1.18 (0.69 - 2.11)	0.549	1.66 (0.83 - 3.70)	0.154	1.82 (0.97 - 3.90)	0.063	1.85 (0.94 - 4.08)	0.074	1.03 (0.80 - 1.34)	0.816
Rate max increase	1.18 (0.54 - 2.53)	0.660	1.06 (0.34 - 2.56)	0.904	1.17 (0.44 - 2.82)	0.731	0.89 (0.41 - 1.80)	0.739	0.50 (0.15 - 1.24)	0.143	0.43 (0.12 - 1.09)	0.078	0.74 (0.23 - 1.71)	0.507	0.98 (0.70 - 1.37)	0.884
Max decrease	1.23 (0.57 - 2.61)	0.590	0.59 (0.18 - 1.51)	0.294	1.69 (0.68 - 4.35)	0.251	1.09 (0.58 - 2.07)	0.789	0.75 (0.31 - 1.65)	0.474	0.85 (0.40 - 1.75)	0.651	1.43 (0.69 - 3.06)	0.328	1.01 (0.75 - 1.36)	0.931
Rate max decrease	0.92 (0.66 - 1.04)	0.189	0.87 (0.37 - 1.27)	0.572	0.72 (0.33 - 1.03)	0.114	0.94 (0.66 - 1.19)	0.613	0.98 (0.62 - 1.33)	0.896	0.94 (0.55 - 1.33)	0.770	0.89 (0.39 - 1.21)	0.551	0.92 (0.81 - 1.05)	0.203

Definitions of exposure windows, exposure measures, plasma osmolarity calculation, neonatal outcomes, adjustments, and effect scaling are as in Table 2.
36w denotes 36 weeks´postmenstrual age. BPD, bronchopulmonary dysplasia; CI, confidence interval; IRR, incidence rate ratio; IVH, intraventricular hemorrhage; MV, mechanical ventilation; NEC, necrotizing enterocolitis; OR, odds ratio; PDA, patent ductus arteriosus; ROP, retinopathy of prematurity.

Supplementary Table S2. Crude associations of early plasma sodium, glucose, and calculated osmolarity measures with neonatal outcomes.

Outcome (events/N)	IVH (11/42)		PDA surgical ligation (5/41)		NEC (6/42)		ROP (17/41)		BPD (17/33)		BPD/death before 36w (25/41)		Death before 36w (8/41)		Duration of MV (N=41)	
Exposure measure	OR (95% CIs)	p	OR (95% CIs)	p	OR (95% CIs)	p	OR (95% CIs)	p	OR (95% CIs)	p	OR (95% CIs)	p	OR (95% CIs)	p	IRR (95% CIs)	p
P-Sodium																
Median	0.46 (0.16 - 1.11)	0.085	1.30 (0.35 - 6.23)	0.721	0.92 (0.74 - 1.13)	0.412	1.71 (0.68 - 5.01)	0.265	1.66 (0.53 - 5.68)	0.386	1.03 (0.40 - 2.58)	0.955	0.39 (0.10 - 1.16)	0.091	1.32 (0.78 - 2.24)	0.306
Peak	0.97 (0.51 - 1.76)	0.921	0.40 (0.08 - 1.13)	0.091	0.99 (0.83 - 1.14)	0.853	1.42 (0.80 - 2.64)	0.238	1.46 (0.74 - 3.18)	0.281	1.52 (0.84 - 3.04)	0.173	1.48 (0.74 - 3.03)	0.265	1.09 (0.78 - 1.52)	0.601
Nadir	0.89 (0.41 - 1.86)	0.757	0.50 (0.13 - 1.57)	0.241	1.04 (0.87 - 1.26)	0.637	0.74 (0.33 - 1.57)	0.436	0.93 (0.39 - 2.14)	0.854	0.99 (0.46 - 2.14)	0.983	1.21 (0.47 - 3.09)	0.684	0.90 (0.58 - 1.40)	0.645
Range peak-nadir	0.89 (0.46 - 1.62)	0.719	0.15 (0.01 - 0.63)	**0.005**	1.01 (0.87 - 1.17)	0.856	1.17 (0.67 - 2.10)	0.573	1.33 (0.70 - 2.67)	0.383	1.49 (0.83 - 2.90)	0.186	1.61 (0.81 - 3.36)	0.170	1.05 (0.75 - 1.45)	0.788
Max Δ48 h	0.91 (0.41 - 1.81)	0.787	0.23 (0.02 - 1.07)	0.066	1.05 (0.88 - 1.24)	0.552	1.39 (0.72 - 2.84)	0.326	1.32 (0.64 - 3.03)	0.453	1.36 (0.69 - 3.01)	0.382	1.26 (0.55 - 2.69)	0.559	1.16 (0.79 - 1.70)	0.463
Max increase	0.81 (0.43 - 1.44)	0.488	0.31 (0.06 - 0.90)	**0.029**	0.98 (0.83 - 1.12)	0.770	1.31 (0.78 - 2.30)	0.309	1.39 (0.76 - 2.73)	0.284	1.49 (0.86 - 2.80)	0.161	1.44 (0.77 - 2.79)	0.250	1.03 (0.76 - 1.40)	0.846
Rate max increase	0.99 (0.60 - 1.32)	0.938	1.06 (0.65 - 1.31)	0.651	1.06 (1.00 - 1.18)	0.051	0.94 (0.59 - 1.16)	0.578	1.10 (0.62 - 2.06)	0.734	1.05 (0.85 - 1.64)	0.645	1.15 (0.93 - 1.57)	0.181	1.10 (0.95 - 1.26)	0.197
Max decrease	1.34 (0.68 - 2.66)	0.387	0.52 (0.13 - 1.36)	0.199	1.09 (0.92 - 1.28)	0.292	1.03 (0.57 - 1.86)	0.917	1.11 (0.57 - 2.26)	0.751	1.30 (0.72 - 2.52)	0.394	1.70 (0.84 - 3.66)	0.137	1.21 (0.86 - 1.70)	0.266
Rate max decrease	0.89 (0.67 - 1.04)	0.256	0.92 (0.18 - 1.17)	0.588	0.99 (0.92 - 1.02)	0.734	1.01 (0.85 - 1.19)	0.907	1.04 (0.88 - 1.28)	0.629	1.04 (0.88 - 1.28)	0.683	1.02 (0.81 - 1.22)	0.801	0.96 (0.87 - 1.06)	0.424
P-Glucose																
Median	1.08 (0.85 - 1.37)	0.522	1.05 (0.68 - 1.50)	0.788	1.10 (0.81 - 1.46)	0.499	1.33 (1.00 - 1.90)	**0.048**	1.34 (0.98 - 2.03)	0.068	1.33 (0.98 - 1.96)	0.070	1.03 (0.73 - 1.41)	0.837	1.15 (0.99 - 1.34)	0.062
Peak	1.04 (0.92 - 1.18)	0.506	1.00 (0.85 - 1.13)	0.984	1.16 (1.00 - 1.36)	0.052	1.03 (0.95 - 1.14)	0.456	1.16 (1.01 - 1.39)	**0.035**	1.17 (1.03 - 1.40)	**0.011**	1.09 (0.99 - 1.22)	0.088	1.06 (1.01 - 1.12)	**0.015**
Nadir	1.23 (0.69 - 2.35)	0.486	1.37 (0.59 - 4.05)	0.486	1.27 (0.62 - 3.00)	0.522	0.96 (0.55 - 1.71)	0.897	2.49 (1.23 - 6.20)	**0.010**	2.87 (1.44 - 7.01)	**0.002**	2.12 (0.94 - 6.26)	0.074	1.32 (0.96 - 1.82)	0.086
Range peak-nadir	1.05 (0.93 - 1.18)	0.429	1.01 (0.86 - 1.13)	0.917	1.16 (1.00 - 1.36)	**0.044**	1.03 (0.95 - 1.13)	0.479	1.22 (1.04 - 1.51)	**0.008**	1.24 (1.07 - 1.53)	**0.002**	1.10 (1.00 - 1.23)	0.056	1.07 (1.02 - 1.12)	**0.006**
Max Δ48 h	1.05 (0.92 - 1.19)	0.473	1.01 (0.84 - 1.16)	0.934	1.16 (1.00 - 1.37)	0.053	1.05 (0.94 - 1.17)	0.397	1.31 (1.08 - 1.72)	**0.003**	1.34 (1.11 - 1.77)	**<0.001**	1.11 (0.99 - 1.27)	0.078	1.08 (1.02 - 1.15)	**0.006**
Max increase	1.04 (0.92 - 1.17)	0.503	1.01 (0.87 - 1.13)	0.897	1.15 (1.00 - 1.34)	0.052	1.03 (0.94 - 1.13)	0.504	1.23 (1.04 - 1.52)	**0.008**	1.23 (1.06 - 1.51)	**0.002**	1.10 (1.00 - 1.22)	0.062	1.07 (1.02 - 1.12)	**0.008**
Rate max increase	1.01 (0.93 - 1.08)	0.807	1.05 (0.96 - 1.15)	0.246	1.03 (0.94 - 1.12)	0.491	1.03 (0.97 - 1.11)	0.337	1.07 (0.99 - 1.19)	0.106	1.06 (0.98 - 1.16)	0.171	1.00 (0.91 - 1.09)	0.934	1.02 (0.98 - 1.07)	0.264
Max decrease	1.10 (0.94 - 1.31)	0.234	1.11 (0.91 - 1.35)	0.303	1.31 (1.08 - 1.73)	**0.006**	1.10 (0.96 - 1.28)	0.178	1.42 (1.12 - 2.02)	**0.001**	1.50 (1.17 - 2.18)	**<0.001**	1.17 (0.99 - 1.40)	0.063	1.14 (1.06 - 1.22)	**<0.001**
Rate max decrease	1.00 (0.92 - 1.08)	0.949	0.99 (0.77 - 1.10)	0.850	1.07 (0.99 - 1.16)	0.078	0.95 (0.84 - 1.04)	0.316	1.37 (1.05 - 2.00)	**0.010**	1.36 (1.07 - 1.98)	**0.004**	1.09 (1.00 - 1.22)	0.055	1.04 (0.99 - 1.09)	0.147
Calculated P-Osmolarity																
Median	0.59 (0.25 - 1.22)	0.160	0.89 (0.28 - 3.46)	0.848	0.72 (0.28 - 1.76)	0.474	2.11 (0.86 - 6.20)	0.109	2.42 (0.79 - 8.59)	0.125	1.27 (0.54 - 3.09)	0.571	0.44 (0.14 - 1.21)	0.110	1.36 (0.83 - 2.22)	0.219
Peak	0.95 (0.55 - 1.59)	0.860	0.57 (0.19 - 1.32)	0.203	1.14 (0.59 - 2.13)	0.686	1.41 (0.84 - 2.53)	0.199	1.75 (0.91 - 3.93)	0.097	1.88 (1.04 - 3.85)	**0.035**	1.72 (0.91 - 3.51)	0.096	1.17 (0.87 - 1.59)	0.294
Nadir	0.76 (0.31 - 1.67)	0.501	0.74 (0.21 - 2.20)	0.597	1.11 (0.40 - 2.88)	0.834	0.83 (0.38 - 1.73)	0.613	0.96 (0.41 - 2.24)	0.917	0.94 (0.44 - 2.01)	0.878	0.96 (0.37 - 2.36)	0.927	0.95 (0.62 - 1.46)	0.817
Range peak-nadir	0.84 (0.46 - 1.41)	0.525	0.40 (0.12 - 1.04)	0.061	1.18 (0.61 - 2.18)	0.605	1.32 (0.76 - 2.43)	0.334	1.85 (0.92 - 4.31)	0.088	2.00 (1.06 - 4.43)	**0.032**	1.78 (0.90 - 3.99)	0.097	1.21 (0.88 - 1.66)	0.246
Max Δ48 h	0.82 (0.39 - 1.48)	0.522	0.72 (0.20 - 1.71)	0.505	1.14 (0.52 - 2.23)	0.713	1.46 (0.82 - 2.83)	0.196	2.07 (0.97 - 5.82)	0.060	2.31 (1.10 - 6.48)	**0.023**	1.64 (0.85 - 3.31)	0.135	1.43 (1.04 - 1.97)	**0.030**
Max increase	0.81 (0.45 - 1.34)	0.413	0.55 (0.20 - 1.25)	0.161	1.06 (0.55 - 1.93)	0.853	1.34 (0.81 - 2.38)	0.260	2.10 (1.07 - 4.89)	**0.029**	2.35 (1.24 - 5.28)	**0.007**	1.90 (1.00 - 4.19)	0.050	1.23 (0.92 - 1.65)	0.162
Rate max increase	1.12 (0.54 - 2.13)	0.746	1.32 (0.49 - 3.00)	0.531	1.45 (0.65 - 3.03)	0.333	1.10 (0.56 - 2.14)	0.775	0.98 (0.49 - 1.97)	0.963	0.92 (0.47 - 1.82)	0.798	0.88 (0.31 - 1.90)	0.763	1.44 (0.99 - 2.09)	0.056
Max decrease	1.12 (0.57 - 2.18)	0.730	0.86 (0.33 - 1.94)	0.729	1.85 (0.83 - 4.37)	0.132	1.21 (0.69 - 2.16)	0.505	1.15 (0.61 - 2.23)	0.663	1.29 (0.73 - 2.40)	0.391	1.52 (0.77 - 3.13)	0.221	1.30 (0.94 - 1.78)	0.108
Rate max decrease	0.89 (0.67 - 1.04)	0.236	1.01 (0.53 - 1.33)	0.948	0.81 (0.43 - 1.06)	0.243	0.98 (0.75 - 1.24)	0.882	1.02 (0.81 - 1.33)	0.840	0.98 (0.78 - 1.28)	0.897	0.95 (0.49 - 1.24)	0.731	1.06 (0.91 - 1.22)	0.461

Definitions of exposure windows, exposure measures, plasma osmolarity calculation, neonatal outcomes, and effect scaling are as in Table 2.
Exact p-values are provided for all estimates.
36w denotes 36 weeks´postmenstrual age. BPD, bronchopulmonary dysplasia; CI, confidence interval; IRR, incidence rate ratio; IVH, intraventricular hemorrhage; MV, mechanical ventilation; NEC, necrotizing enterocolitis; OR, odds ratio; PDA, patent ductus arteriosus; ROP, retinopathy of prematurity.

Supplementary Table S3. Associations of early plasma sodium, glucose, and calculated osmolarity measures with duration of mechanical ventilation among infants surviving to discharge.

Exposure measure	Crude IRR (95% CIs)	p	Adjusted[a] IRR (95% CIs)	p
P-Sodium				
Median	1.30 (0.63 - 2.68)	0.486	0.92 (0.51 - 1.64)	0.773
Peak	1.07 (0.69 - 1.66)	0.751	0.87 (0.62 - 1.22)	0.419
Nadir	0.83 (0.49 - 1.43)	0.504	0.79 (0.51 - 1.22)	0.289
Range peak-nadir	1.00 (0.66 - 1.50)	0.983	0.78 (0.57 - 1.08)	0.138
Max Δ48 h	1.11 (0.70 - 1.78)	0.654	0.74 (0.51 - 1.08)	0.122
Max increase	0.98 (0.67 - 1.44)	0.929	0.83 (0.61 - 1.13)	0.229
Rate max increase	1.32 (0.91 - 1.90)	0.145	0.88 (0.63 - 1.22)	0.438
Max decrease	1.20 (0.78 - 1.86)	0.398	0.89 (0.62 - 1.29)	0.545
Rate max decrease	0.96 (0.86 - 1.08)	0.535	0.95 (0.86 - 1.05)	0.353
P-Glucose				
Median	1.15 (0.96 - 1.37)	0.128	1.03 (0.88 - 1.22)	0.686
Peak	1.09 (1.02 - 1.16)	**0.016**	1.03 (0.97 - 1.10)	0.267
Nadir	1.40 (0.96 - 2.05)	0.080	1.67 (1.26 - 2.21)	**<0.001**
Range peak-nadir	1.10 (1.03 - 1.17)	**0.005**	1.05 (0.99 - 1.11)	0.110
Max Δ48 h	1.10 (1.03 - 1.19)	**0.008**	1.06 (0.99 - 1.13)	0.071
Max increase	1.10 (1.03 - 1.17)	**0.006**	1.05 (0.99 - 1.11)	0.108
Rate max increase	1.03 (0.98 - 1.08)	0.299	1.02 (0.98 - 1.07)	0.275
Max decrease	1.17 (1.07 - 1.28)	**0.001**	1.10 (0.99 - 1.21)	0.071
Rate max decrease	1.09 (1.01 - 1.18)	**0.028**	1.04 (0.97 - 1.11)	0.290
Calculated P-Osmolarity				
Median	1.43 (0.71 - 2.87)	0.315	1.02 (0.58 - 1.80)	0.946
Peak	1.20 (0.81 - 1.78)	0.375	0.97 (0.70 - 1.35)	0.873
Nadir	0.89 (0.52 - 1.53)	0.675	0.90 (0.58 - 1.40)	0.634
Range peak-nadir	1.26 (0.82 - 1.93)	0.289	0.90 (0.63 - 1.30)	0.581
Max Δ48 h	1.55 (1.01 - 2.36)	**0.044**	0.83 (0.56 - 1.22)	0.340
Max increase	1.32 (0.89 - 1.93)	0.163	0.94 (0.68 - 1.30)	0.714
Rate max increase	1.37 (0.89 - 2.11)	0.147	0.87 (0.60 - 1.28)	0.487
Max decrease	1.31 (0.88 - 1.96)	0.181	0.93 (0.65 - 1.33)	0.681
Rate max decrease	1.05 (0.89 - 1.23)	0.553	0.92 (0.80 - 1.05)	0.224

Exposure window was defined as the first 7 postnatal days; analyses included 33 survivors to discharge with eligible 7-day exposure data (one survivor excluded due to abdominal surgery on day 5).

Definition of exposure measures, plasma osmolarity calculation, effect estimates and scaling are as in Table 2.

IRR was estimated by negative binomial regression.

[a] Adjusted for gestational age entered as a standardized z-score (calculated using the mean and SD of gestational age in the full study cohort), birth weight z-score, and sex.

CI, confidence interval; IRR, incidence rate ratio

Supplementary Table S4. Sensitivity analysis of the associations of early plasma sodium, glucose, and calculated plasma osmolarity measures with neonatal outcomes after additional adjustment for maximum percentage weight loss from birth weight during the exposure window.

Outcome (events/N)	IVH (11/42)		PDA surgical ligation (5/41)		NEC (6/42)		ROP (17/41)		BPD (17/33)		BPD/death before 36w (25/41)		Death before 36w (8/41)		Duration of MV (N=41)	
Exposure measure	OR (95% CIs)	p	OR (95% CIs)	p	OR (95% CIs)	p	OR (95% CIs)	p	OR (95% CIs)	p	OR (95% CIs)	p	OR (95% CIs)	p	IRR (95% CIs)	p
P-Sodium																
Median	0.59 (0.21 - 1.46)	0.260	1.11 (0.23 - 6.07)	0.895	0.86 (0.24 - 2.68)	0.795	1.59 (0.54 - 5.17)	0.401	2.10 (0.55 - 10.22)	0.278	0.93 (0.29 - 2.92)	0.905	0.46 (0.11 - 1.45)	0.191	0.86 (0.54 - 1.37)	0.528
Peak	1.10 (0.56 - 2.17)	0.773	0.39 (0.07 - 1.06)	0.067	1.00 (0.45 - 2.15)	0.993	1.41 (0.77 - 2.77)	0.269	1.45 (0.63 - 3.92)	0.399	1.58 (0.72 - 3.99)	0.262	1.48 (0.74 - 3.22)	0.268	0.91 (0.69 - 1.21)	0.525
Nadir	0.62 (0.21 - 1.57)	0.325	0.53 (0.11 - 1.71)	0.307	1.05 (0.30 - 2.92)	0.936	0.71 (0.29 - 1.66)	0.428	0.81 (0.25 - 2.43)	0.704	0.95 (0.36 - 2.59)	0.913	1.06 (0.37 - 2.79)	0.910	1.02 (0.70 - 1.49)	0.914
Range peak-nadir	0.88 (0.44 - 1.62)	0.680	0.18 (0.02 - 0.62)	**0.004**	1.01 (0.45 - 2.11)	0.987	1.17 (0.65 - 2.20)	0.605	1.21 (0.59 - 2.66)	0.605	1.40 (0.71 - 3.01)	0.339	1.50 (0.76 - 3.19)	0.245	0.91 (0.69 - 1.20)	0.506
Max Δ48 h	0.86 (0.37 - 1.80)	0.702	0.19 (0.02 - 0.85)	**0.026**	1.11 (0.43 - 2.65)	0.816	1.29 (0.64 - 2.79)	0.475	1.07 (0.46 - 2.72)	0.868	1.13 (0.50 - 2.72)	0.762	1.11 (0.47 - 2.46)	0.793	0.84 (0.60 - 1.16)	0.283
Max increase	0.83 (0.43 - 1.50)	0.549	0.28 (0.05 - 0.84)	**0.020**	0.87 (0.38 - 1.77)	0.714	1.26 (0.73 - 2.30)	0.413	1.33 (0.69 - 2.75)	0.395	1.42 (0.78 - 2.78)	0.249	1.40 (0.73 - 2.86)	0.309	0.92 (0.71 - 1.19)	0.517
Rate max increase	1.05 (0.53 - 1.47)	0.793	1.01 (0.48 - 1.27)	0.922	1.41 (1.00 - 2.45)	0.052	0.90 (0.43 - 1.14)	0.432	0.65 (0.26 - 1.38)	0.263	1.00 (0.80 - 1.34)	0.995	1.17 (0.93 - 1.56)	0.155	1.01 (0.89 - 1.13)	0.931
Max decrease	1.35 (0.62 - 2.97)	0.442	0.45 (0.11 - 1.17)	0.108	1.22 (0.46 - 2.96)	0.667	0.98 (0.51 - 1.89)	0.953	0.74 (0.29 - 1.78)	0.510	0.99 (0.45 - 2.15)	0.977	1.57 (0.75 - 3.53)	0.234	1.07 (0.79 - 1.44)	0.678
Rate max decrease	0.93 (0.66 - 1.04)	0.246	0.79 (0.11 - 1.16)	0.348	0.98 (0.65 - 1.09)	0.701	1.00 (0.81 - 1.20)	0.963	1.09 (0.89 - 1.34)	0.366	1.06 (0.87 - 1.29)	0.513	1.02 (0.79 - 1.23)	0.810	0.94 (0.86 - 1.03)	0.177
P-Glucose																
Median	1.21 (0.92 - 1.69)	0.176	0.88 (0.54 - 1.29)	0.516	1.13 (0.82 - 1.57)	0.448	1.18 (0.86 - 1.69)	0.312	1.09 (0.73 - 1.67)	0.653	1.10 (0.71 - 1.68)	0.681	1.04 (0.71 - 1.50)	0.830	1.06 (0.91 - 1.22)	0.475
Peak	1.09 (0.94 - 1.30)	0.247	0.94 (0.74 - 1.08)	0.428	1.16 (0.98 - 1.45)	0.079	0.99 (0.89 - 1.09)	0.849	1.05 (0.93 - 1.29)	0.448	1.09 (0.97 - 1.33)	0.178	1.12 (1.00 - 1.28)	**0.045**	1.03 (0.99 - 1.08)	0.161
Nadir	1.00 (0.55 - 1.90)	0.997	1.64 (0.65 - 6.24)	0.324	1.03 (0.48 - 2.41)	0.950	1.08 (0.58 - 2.09)	0.813	3.36 (1.37 - 14.27)	**0.005**	4.67 (1.74 - 23.42)	**<0.001**	1.79 (0.79 - 5.69)	0.178	1.70 (1.32 - 2.19)	**<0.001**
Range peak-nadir	1.08 (0.94 - 1.27)	0.265	0.96 (0.78 - 1.09)	0.578	1.15 (0.98 - 1.40)	0.086	0.99 (0.90 - 1.09)	0.887	1.12 (0.96 - 1.44)	0.185	1.18 (1.00 - 1.51)	**0.047**	1.13 (1.01 - 1.27)	**0.034**	1.04 (1.00 - 1.09)	0.056
Max Δ48 h	1.08 (0.94 - 1.27)	0.278	0.95 (0.76 - 1.11)	0.563	1.15 (0.98 - 1.42)	0.086	1.00 (0.89 - 1.12)	0.994	1.21 (0.99 - 1.60)	0.072	1.27 (1.04 - 1.70)	**0.015**	1.14 (1.00 - 1.31)	0.055	1.06 (1.01 - 1.12)	**0.019**
Max increase	1.07 (0.93 - 1.24)	0.329	0.96 (0.79 - 1.10)	0.609	1.14 (0.98 - 1.37)	0.099	0.99 (0.90 - 1.09)	0.851	1.12 (0.96 - 1.46)	0.169	1.18 (1.00 - 1.52)	**0.045**	1.12 (1.01 - 1.26)	**0.038**	1.04 (1.00 - 1.09)	0.055
Rate max increase	1.02 (0.94 - 1.10)	0.577	1.04 (0.95 - 1.14)	0.369	1.04 (0.94 - 1.13)	0.454	1.03 (0.96 - 1.11)	0.452	1.05 (0.96 - 1.18)	0.253	1.04 (0.96 - 1.15)	0.315	1.01 (0.91 - 1.09)	0.887	1.02 (0.99 - 1.05)	0.245
Max decrease	1.18 (0.97 - 1.50)	0.090	1.02 (0.81 - 1.27)	0.871	1.37 (1.08 - 2.07)	**0.008**	1.03 (0.87 - 1.23)	0.716	1.31 (1.00 - 1.88)	0.051	1.35 (1.05 - 1.90)	**0.016**	1.23 (0.99 - 1.69)	0.064	1.08 (1.00 - 1.17)	**0.038**
Rate max decrease	1.03 (0.94 - 1.12)	0.467	0.90 (0.59 - 1.06)	0.309	1.11 (1.02 - 1.25)	**0.022**	0.90 (0.73 - 1.01)	0.087	1.15 (0.95 - 1.90)	0.210	1.30 (0.99 - 1.98)	0.066	1.12 (1.01 - 1.28)	**0.037**	1.01 (0.96 - 1.06)	0.701
Calculated P-Osmolarity																
Median	0.73 (0.31 - 1.62)	0.443	0.62 (0.14 - 2.73)	0.515	0.87 (0.30 - 2.35)	0.783	2.03 (0.70 - 6.73)	0.197	2.57 (0.69 - 13.09)	0.162	1.05 (0.35 - 3.22)	0.933	0.49 (0.14 - 1.45)	0.204	0.91 (0.59 - 1.42)	0.685
Peak	1.08 (0.60 - 1.96)	0.783	0.47 (0.14 - 1.09)	0.082	1.18 (0.61 - 2.34)	0.617	1.26 (0.71 - 2.32)	0.433	1.55 (0.68 - 4.10)	0.311	1.82 (0.85 - 4.44)	0.128	1.87 (0.93 - 4.40)	0.080	0.98 (0.75 - 1.28)	0.873
Nadir	0.47 (0.14 - 1.24)	0.133	0.85 (0.24 - 2.60)	0.769	0.80 (0.21 - 2.34)	0.701	0.88 (0.37 - 2.11)	0.767	0.96 (0.30 - 3.03)	0.941	0.97 (0.35 - 2.84)	0.952	0.75 (0.25 - 1.97)	0.570	1.16 (0.79 - 1.71)	0.449
Range peak-nadir	0.84 (0.45 - 1.45)	0.542	0.21 (0.02 - 0.74)	**0.011**	1.10 (0.54 - 2.10)	0.779	1.21 (0.66 - 2.37)	0.548	1.45 (0.67 - 3.47)	0.347	1.57 (0.81 - 3.38)	0.184	1.63 (0.82 - 3.63)	0.162	1.03 (0.78 - 1.37)	0.818
Max Δ48 h	0.82 (0.38 - 1.54)	0.552	0.30 (0.03 - 1.21)	0.110	1.01 (0.42 - 2.10)	0.976	1.27 (0.68 - 2.68)	0.464	1.33 (0.58 - 3.62)	0.506	1.53 (0.74 - 4.04)	0.264	1.55 (0.77 - 3.23)	0.215	1.05 (0.78 - 1.42)	0.737
Max increase	0.82 (0.45 - 1.40)	0.477	0.34 (0.08 - 0.95)	**0.038**	1.00 (0.49 - 1.87)	0.995	1.18 (0.68 - 2.18)	0.551	1.59 (0.80 - 3.51)	0.188	1.80 (0.97 - 3.80)	0.062	1.90 (0.96 - 4.41)	0.065	1.04 (0.80 - 1.34)	0.782
Rate max increase	1.08 (0.43 - 2.40)	0.861	1.08 (0.35 - 2.50)	0.877	1.02 (0.32 - 2.74)	0.975	0.91 (0.43 - 1.82)	0.780	0.56 (0.18 - 1.27)	0.173	0.45 (0.13 - 1.11)	0.086	0.69 (0.21 - 1.69)	0.447	0.98 (0.70 - 1.37)	0.893
Max decrease	1.15 (0.53 - 2.50)	0.710	0.60 (0.17 - 1.53)	0.308	1.62 (0.65 - 4.20)	0.292	1.07 (0.56 - 2.07)	0.832	0.73 (0.30 - 1.61)	0.442	0.85 (0.40 - 1.76)	0.663	1.41 (0.68 - 3.00)	0.344	1.02 (0.76 - 1.37)	0.902
Rate max decrease	0.93 (0.68 - 1.05)	0.283	0.87 (0.38 - 1.27)	0.554	0.69 (0.26 - 1.05)	0.164	0.94 (0.64 - 1.23)	0.679	1.00 (0.60 - 1.40)	0.995	0.93 (0.56 - 1.32)	0.722	0.89 (0.38 - 1.20)	0.539	0.92 (0.81 - 1.05)	0.213

Definitions of exposure windows, exposure measures, osmolarity calculation, neonatal outcomes, adjustments (with the addition of maximum percentage weight loss), and effect scaling are as in Table 2.
36w denotes 36 weeks´ postmenstrual age. BPD, bronchopulmonary dysplasia; CI, confidence interval; IRR, incidence rate ratio; IVH, intraventricular hemorrhage; MV, mechanical ventilation; NEC, necrotizing enterocolitis; OR, odds ratio; PDA, patent ductus arteriosus; ROP, retinopathy of prematurity.
